# Limits of Principal Components Analysis for Producing a Common Trait Space: Implications for Inferring Selection, Contingency, and Chance in Evolution

**DOI:** 10.1371/journal.pone.0007957

**Published:** 2009-11-23

**Authors:** Kevin J. Parsons, W. James Cooper, R. Craig Albertson

**Affiliations:** Department of Biology, Syracuse University, Syracuse, New York, United States of America; Michigan State University, United States of America

## Abstract

**Background:**

Comparing patterns of divergence among separate lineages or groups has posed an especially difficult challenge for biologists. Recently a new, conceptually simple methodology called the “ordered-axis plot” approach was introduced for the purpose of comparing patterns of diversity in a common morphospace. This technique involves a combination of principal components analysis (PCA) and linear regression. Given the common use of these statistics the potential for the widespread use of the ordered axis approach is high. However, there are a number of drawbacks to this approach, most notably that lineages with the greatest amount of variance will largely bias interpretations from analyses involving a common morphospace. Therefore, without meeting a set of *a priori* requirements regarding data structure the ordered-axis plot approach will likely produce misleading results.

**Methodology/Principal Findings:**

Morphological data sets from cichlid fishes endemic to Lakes Tanganyika, Malawi, and Victoria were used to statistically demonstrate how separate groups can have differing contributions to a common morphospace produced by a PCA. Through a matrix superimposition of eigenvectors (scale-free trajectories of variation identified by PCA) we show that some groups contribute more to the trajectories of variation identified in a common morphospace. Furthermore, through a set of randomization tests we show that a common morphospace model partitions variation differently than group-specific models. Finally, we demonstrate how these limitations may influence an ordered-axis plot approach by performing a comparison on data sets with known alterations in covariance structure. Using these results we provide a set of criteria that must be met before a common morphospace can be reliably used.

**Conclusions/Significance:**

Our results suggest that a common morphospace produced by PCA would not be useful for producing biologically meaningful results unless a restrictive set of criteria are met. We therefore suggest biologists be aware of the limitations of the ordered-axis plot approach before employing it on their own data, and possibly consider other, less restrictive methods for addressing the same question.

## Introduction

Determining the relative contributions of natural selection, historical contingency, and chance events in evolutionary radiations has been a longstanding challenge in biology, especially from a quantitative perspective. In a recent article from PLoS One [1], Young et al. introduce a modified methodology of principal components analysis (PCA) combined with linear regression called ‘ordered-axis plots’ to test whether radiations of African rift lake cichlids display differences in diversity and patterns of convergence, or non-convergence centered around a common mean. Using this method a single PCA is first carried out on equally sized groups simultaneously in order to create a common trait space, secondly PC scores on each axis are ordered from highest to lowest for each group, and third ordered axes are plotted and tested for differences in slope (indicating differences in variance) using linear regression. The authors make a compelling case from their analysis that African cichlids have evolved along similar axes, and that diversity is age-ordered with lower diversity existing in the youngest radiation from Lake Victoria. Although this study may appear methodologically appealing given the ease with which PCA and linear regression can be combined to produce the ‘ordered-axis plot’ approach, we feel it is important to highlight the major limitations this method introduces that can lead to inaccurate conclusions about patterns of evolutionary diversification.

**Figure 1:**
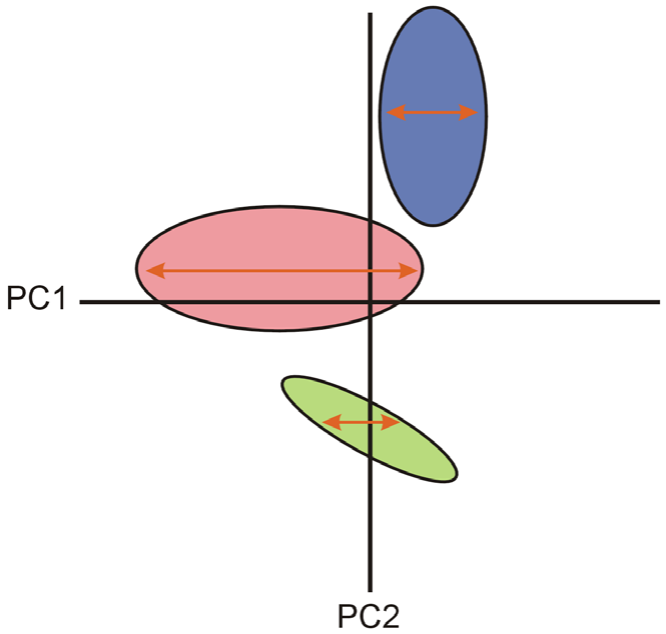
In a common morphospace, major axes of morphological diversity may still differ among groups of interest. Ordered axis plots may not be able to discriminate between patterns of morphological diversity along axes of a multidimensional morphospace because of its reliance upon principal components analysis and the inherent biases of this method. Here the aspects of diversity parallel to PC1 are highlighted with a red arrow for each group of interest. Note that the length of most variable group (pink) is parallel to PC1 because it has the greatest influence over the determination of PC1 in this common morphospace. Other less variable groups (blue, green) have less influence over the trajectory of PC1, but still possess variation that lies parallel to PC1. However the greatest axis of variation within these less variable groups may lie along a vector that differs from PC1. Without knowing a priori whether axes of variation among distinct groups are similar, it is impossible to know the degree to which an ordered axis plots approach will yield misleading results.

PCA is one of the more straightforward multivariate methods and is primarily used to reduce dimensionality in data sets by ‘concentrating’ variation into fewer uncorrelated variables. This process relies on identifying eigenvectors, the scale-free trajectories that describe the maximum covariance or correlations among variables. For evolutionary studies eigenvectors may identify primary trajectories of divergence. PCA is most efficient at reducing dimensionality when the original variables are highly correlated, allowing the majority of variation to be explained by just a few vectors [2], [3]. This means that variables that possess higher degrees of both variance and associated covariance will have a greater influence over how PC axes (PCs) are determined. In other words, in a pooled analysis the major axis of divergence in a more variable group may ‘swamp’ the vectors present in other less variable groups, making it appear as though all groups are diverging the same way (Figure 1.). This influence is further enhanced by the requirement of orthogonality (lack of correlation) among PC axes. PC1, for example, accounts for the greatest degree of variation, and will influence the direction of all subsequent PCs because they must be orthogonal to this first axis [2], [3]. To alleviate this problem a PCA can be performed on a scale-free correlation matrix rather than a covariance matrix, but outliers could still have a strong influence in defining the direction of the first PC.

In practice this means that a PCA applied to several groups simultaneously, as occurs in the ‘ordered-axis plots approach’, may not accurately account for variation in groups displaying relatively lower magnitudes of covariance among traits (Figure 1). In turn, PCs created from this method may not accurately describe the major trajectories of evolution specific to less variable groups. Although having equal sample sizes may alleviate this issue somewhat, as is the case in Young et al. [1], variance and covariance are not a function of sample size.

## Methods

**Figure 2:**
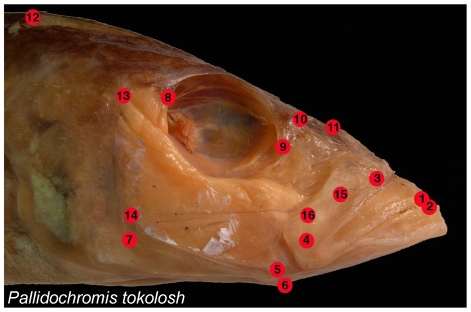
Anatomical landmarks examined: 1 = Tip of the anterior-most tooth on the premaxilla; 2 = Tip of the anterior-most tooth on the dentary; 3 = Maxillary-palatine joint (upper rotation point of the maxilla); 4 = Maxillary-articular joint (lower point of rotation of the maxilla); 5 = Articular-quadrate joint (lower jaw joint); 6 = Insertion of the interopercular ligament on the articular (point at which moth opening forces are applied); 7 = Posterio-ventral corner of the preopercular; 8 = Most posterio-ventral point of the eye socket; 9 = The most anterio-ventral point of the eye socket; 10 = Joint between the nasal bone and the neurocranium; 11 = Posterior tip of the ascending process of the premaxilla; 12 = Dorsal-most tip of the supraoccipital crest on the neurocranium; 13 = Most dorsal point on the origin of the A1 division of the *adductor mandibulae* jaw closing muscle on the preopercular; 14 = Most dorsal point on the origin of the A12 division of the *adductor mandibulae* jaw closing muscle on the preopercular; 15 = Insertion of the A1 division of the *adductor mandibulae* on the maxilla; 16 = Insertion of the A2 division of the *adductor mandibulae* on the articular process.

Here we demonstrate the potentially confounding effects of these problems using our own geometric morphometric data set of cichlid craniofacial shape from each of the three African rift lake assemblages (Figure 2.). To begin we performed a common translation, rotation, and scaling of size on our complete set of landmark coordinates [3]. Partial warp scores (shape variables), including uniform scores, were obtained from these aligned coordinates and were then used in a PCA of each lake assemblage separately, and a PCA on all lakes combined. The combined PCA represented a common morphospace for all cichlids similar to what was calculated in Young et al. [1].

## Results and Discussion

**Figure 3:**
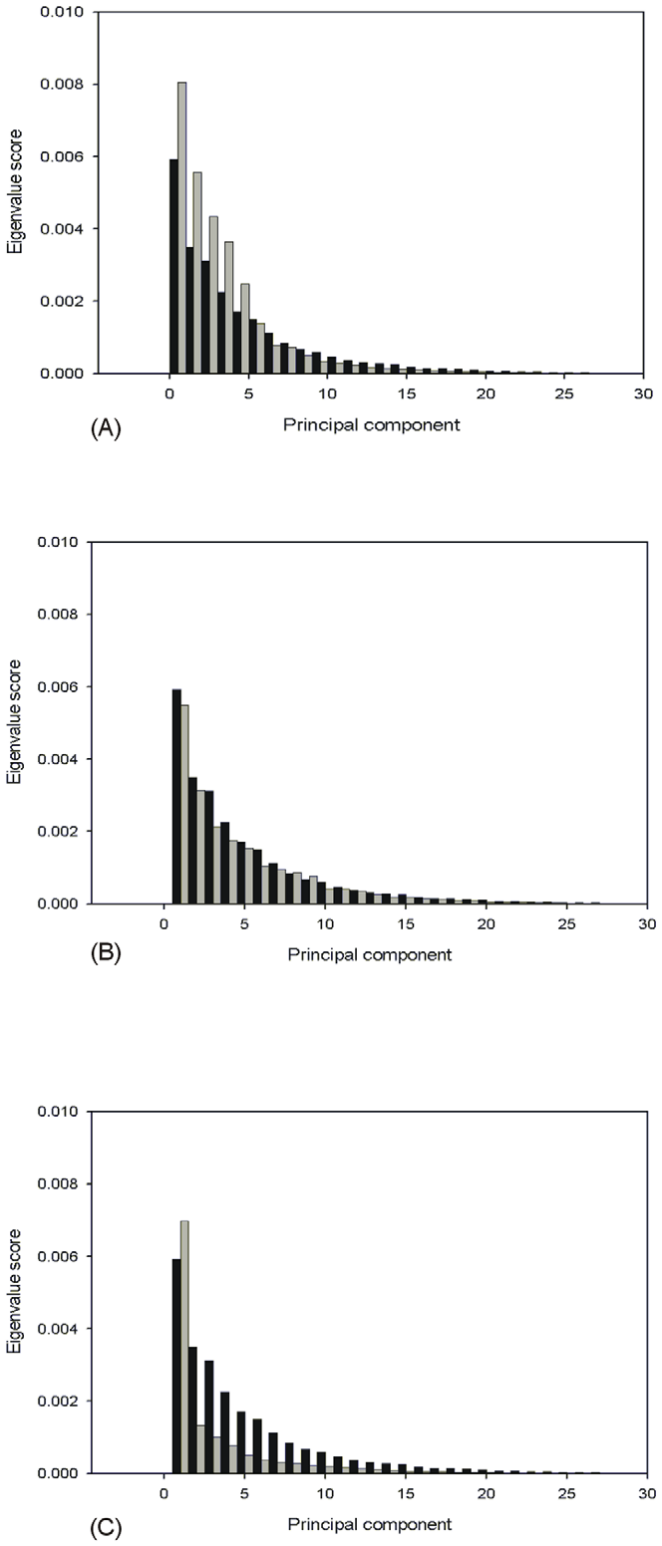
Scree plots showing the spread of craniofacial shape variation across the eigenvalues for the common morphspace (black bars repeated in each plot) and each of the African rift lake cichlid assemblages (grey bars). Tanganyika is represented in (A), Malawi in (B) and Victoria in (C).

We predicted that primary vectors of cichlid divergence identified by a PCA within lakes would differ from those identified by a PCA run on the combined dataset from all lakes (Figure 2.). We therefore tested for differences in the variance of eigenvalues from different PCA models. Eigenvalues are a scalar value used to represent the amount of variation each eigenvector accounts for in a given PCA [2], [3]. If covariation among traits is high, the first few PCs present large eigenvalues relative to later ones, and the variance of eigenvalues is high. If covariation is low, PCs have similar eigenvalues and variance among them is low [4], [5]. We used a procedure that bootstrapped the differences in eigenvalue variance 1000 times by sampling with replacement from rows of our raw data [6], and found that Tanganyika displayed significantly higher variance in eigenvalues compared to the common morphospace (σ^2^ = 0.011 versus 0.008 respectively, p = 0.004). This suggested that the common morphospace model did not accurately reflect the patterns of trait covariation found in Lake Tanganyika (Figure 3A). Without this investigation we would only be able to assume that variation in cichlid traits was spread similarly across PCs in each of the three lakes. There were no significant differences in the spread of eigenvalue variance in comparisons of both Malawi and Victoria to the common morphospace (Figure 3B,C).

In addition to testing for incongruent eigenvalue variances, we were interested in determining whether the vectors of divergence identified in common morphospace accurately reflected lake specific directions of cichlid divergence. To determine whether the primary directions of divergence differed between the common morphospace and each lake we extracted the first 5 eigenvectors from each of our PCA models for use in Procrustes matrix superimpositions [7], [8]. Eigenvectors are orthogonal and summarize information about data covariation independent of scale, and so were useful for comparisons of vector direction among the PCA models describing cichlid evolution. Procrustes matrix superimpositions are a method of matrix correlation that allows for tests of association using raw untransformed data [7]. The concordance of two eigenvector matrices (i.e. lake specific vectors versus the common morphospace vectors) can then be determined and tested based on a goodness-of-fit measure. The sum of the squared residuals between eigenvector matrices provides a goodness-of-fit statistic (m12) that ranges between 0 and 1, and identifies the optimal superimposition that can be used as a metric of concordance. Small values of m12 correspond to small residual variation and, hence, a high concordance of matrices.

Our tests of association using 1000 bootstrapped replicates revealed that the eigenvectors of the Victoria PCA model were not significantly associated with the common morphospace PCA (p = 0.072, m12 = 0.796). While eigenvectors in Tanganyika and Malawi were significantly associated with the common PCA (p<0.01, m12 = 0.6371; and p<0.01, m12 = 0.5476, respectively), high m12 values suggested that the concordance was not strong. Taken together these results suggested that Malawi and Tanganyika had a greater influence over the calculation of the common morphospace than Victoria, and that the common morphospace also had vectors that largely did not align with the vectors identified independently in each lake. This problem was exacerbated when we extended our analysis to include scale with our eigenvectors by investigating potential associations between the first 5 PCs of the common morphospace, and the first 5 lake specific PCs. Both Victoria and Tanganyika had no association between PC axes (p = 1.0, m12 = 0.991; p = 0.081, m12 = 0.874 respectively), while the m12 value increased in Malawi (p<0.01, m12 = 0.624). Therefore, in this analysis our common morphospace did not correspond well to the major axes of variation identified in the different lake assemblages.

To explicitly demonstrate how these biases could affect an ordered-axis plots analysis we tested its performance on data with known alterations in covariance structure. We first removed the effect of PC1 from the original raw landmark data used to create the above common cichlid morphospace using a multiple regression in the program Standard 6 [9]. Thus, variation identified by PC1, which accounted for more than 23.7% of the variance in our original PCA, was now largely absent from the data set. We next used both this PC1 standardized data set and the original data as groups for comparison in an ordered axis plot approach [1]. This would be akin to comparing biological groups that differ in both their levels of variation, and their primary direction of variation, which should bias any analysis in a common trait space. However, the ordered-axis plot approach revealed a regression slope of 0.98 on the new PC1, and an intercept of 0.02 (slope of 1 indicates equal levels of variance between groups, intercept of 0 indicates a common trajectory of divergence), indicating that the major axes of variation, and levels of variation between these data sets were extremely similar. This result is striking because we knowingly removed the major axis of variation from one of the data sets. Any interpretation of biological processes such as historical contingency or selection derived from this type of analysis would therefore be highly questionable.

Although these results highlight the potential problems of interpreting data from a common PCA on multiple groups we do not feel they have been especially detrimental to the results of Young et al. [1]. In fact their main conclusion of a common axis of divergence is supported by our own data (analysis not shown). However, in their analysis of lake-specific morphospace, where the angles of PCs were compared between lakes, differences did exist between Tanganyika and Victoria for total body shape on PC1 (i.e., M_max_), which makes any interpretation from the ordered-axis plots approach (i.e., combined morphospace) questionable for total body shape in these two assemblages (see Figure 3. in Young et al., [1]). Furthermore, Young et al. [1] provide no comparison of the common PCA model to the lake specific models, making it difficult to know if their common morphospace accurately reflects the major axes of divergence in each lake. Lastly, the calculations of slope and intercept found in table 1 of Young et al. [1] would benefit greatly from the generation of confidence intervals from a resampling procedure to determine whether their values differ from random. In their present form the values used to generate rankings in table 1 do not indicate any statistical significance that allows us to reliably determine whether evolutionary trajectories, or variation differs among cichlid assemblages.

The ordered-axis plot method is conceptually appealing and methodologically straight forward, but we feel that it will only be of limited use to biologists interested in understanding the repeatability of evolutionary radiations if their data meet the following criteria:

1. Sample sizes among groups are equal [1].
2. The direction of evolution (covariance) is the same in all groups.

It is the this second criterion that is especially important for producing accurate results from an ordered-axis plot approach, because, as we have shown, particular groups can bias a common morphospace (Figure 1.). There are several methods for testing whether the direction of evolution is statistically similar across groups, including the methods we have used here, common principal components analysis [10], [11], comparisons of PCA subspace [1] or other methods of trajectory comparison [12]. Our findings suggest the application of ordered-axis plots is only useful for confirming, not discovering, common trajectories of evolution. It is a method that is probably more useful for testing differences in the mean and variances of groups along specific, constrained axes of morphospace. It is worth pointing out however, that several traditional tests like ANOVA and F-tests for homogeneity of variance already exist, and are well suited to this task, as shown by their use in Young et al. [1]. In addition, while it may be of interest to look at divergence on specific axes in some studies, morphometric methods have existed for several years that allow for tests of differences in means and variance in a wholly unconstrained shape space [3], [13], [14]. Given the myriad of time-tested methods that are available for examining patterns of divergence, and the limitations of the ordered-axis plot approach, we urge biologists to be thoughtful when considering this technique.
